# 

**Published:** 1800-11

**Authors:** 


### NEW MEDICAL PUBLICATIONS IN OCTOBER.

A Treatiſe on Febrile Diſeaſes, including intermitting, remitting, and continued Fevers; eruptive Fevers; Inflammations; Hæmorrhagies and the Profluvia; in which an attempt is made to preſent, at one view, whatever in the preſent ſtate of medicine it is requiſite for the Phyſician to know, reſpecting the ſymptoms, cauſes, and cure of thoſe diſeaſes. By A. Philips Wilſon, M. D. F. R. S. Ed. &c. 8vo. 9s. Cadell and Davies, Callow, &c.

A View of the moſt important Facts which have appeared concerning the Inoculation for the Cow-pox, by C. R. Aikin, Surgeon, with a coloured Plate, repreſenting the Puſtules in different Stages, 2s. 6d. Phillips.

An Appendix to the Treatiſes on the Cow-pox, being a Continuation of Facts and Obſervations relating to that Diſeaſe, by Edward Jenner, M. D. 2s. 6d. Low.

The Hoſpital Pupil's Guide, with Anecdotes relative to the Hiſtory and Economy of Hoſpitals, 2s. Weſt and Hughes.

A conciſe View of Circumſtances and Proceedings reſpecting Vaccine Inoculation, &c. 2s. Hurſt.

The Naval Guardian, by Charles Fletcher, M. D. Author of "A Maritime State conſidered as to the State of Health of Seamen, &c." 2 vols. 14s, boards. Chapman.

### NEW MEDICAL PUBLICATIONS IN FRANCE.

*Memoires de Medicine Pratique, &c.* i. e. Memoirs of Practical Medicine; on the Climate and Diſeaſes of the Mantouan Territory, on Peruvian Bark, on the frequent Cauſes of Chronic Diarrhœas in young Soldiers, and on the preſent Epidemic of Nice. By F. E. Foderé, D. M. 1 vol. 8vo. year 8, 1800, à Paris.

*Traité de Membranes,* i. e. Treatiſe on Membranes in general, and on different Membranes in particular. By Xavier Bichat, of the Society of Medicine, &c. 8vo. year 8. Paris, chez Richard, &c.

*Leçons d'Anatomie comparée, &c.* i. e. Lectures on Comparative Anatomy of Cit. Cuvier, of the National Inſtitute, &c. Collected and publiſhed under his inſpection by Cit. Dumeril, &c. 2 vol. in 8vo. pp. 500, enriched with engravings. Paris, for Baudouin.

### NEW MEDICAL PUBLICATIONS IN GERMANY.

*Die Werkzeuge,* i. e. The Inſtruments of Ancient and Modern Obſtetric Art. By B. N. G. Schreger, Prof. at Erlangen. T. 1. fol. 1799. Erlangen, for Palm. Price 1 rixd. or about 3s. 6d.

*Soemmering Tabulae baſeos Encephali.* Fol. Frankfort, 1800, price 2 rixd. 12 gr. or about 9s. 6d.

Archiv

*Archiv fur Zoologie*, i. e. Archives for Zoology and Zootomy, publifhed by Wiedeman, Prof. at Brunfwic, Vol. I. No. 1. with plates, 1800. Berlin, for Vofs. 8vo. price 2 rixd. or about 3s. 6d.

*Annalen*, i. e. Annals of the lateft Englifh and French Surgery and Midwifery; edited by Prof. SCHREGER and HARLES, Vol. I. No. I. II. III. with plates, 1800, price 18 grofehen, or about 2s. 6d. each number. Erlangen, Schubart.

*C. W. Bockmann's Phyfiche Chemifche Verfuche, &c.* i. e. Phyfico-Chemical Experiments on Phofphorus in different Species of Gafes, particularly in Azote and Oxygen Gas, with a Preface, and fome Notes of Prof. Hildebrandt; with plates, 1800, 8vo. price 1 rixd. 12 gr. or about 5s. Erlangen, Schubart.

*Callisen Henr. Principia Syftematis Chirurgiae Hodiernae, Pars pofterior*, edit. nova, 1800, price 2 rixd. 8 gr. or about 8s. Hafnia, Proft.

*Karften's, L. G. Mineralogifche Tabellen*, i. e. Mineralogical Tables, compofed according to the lateft Difcoveries, fol. 1800, price 1 rixd. 8 gr. or about 5s. Berlin, Rottman.

*Links, J. W. Grundalze der Pharmazie, &c.* i. e. Elements of Pharmacy, together with its Hiftory and Literature, 8vo. 1800, Vol. I. p. I. et II. price 3 rixd. or about 10s.

*Ophthalmologia Pathologica, S. de Cognofcendis et Curandis Organi vifarii Affectionibus Liber.* Sect. I. 8vo. 1800, price 20 gr. or about 3s. Lipfiae, Fleifher, jun.

*Retzii, A. T. Fauna Suecica*, 8vo. 1800, price 1 rixd. 12 gr. or about 5s. 6d. Lipfiae, Crufius.

### To CORRESPONDENTS.

DR. BRADLEY, *to whom alone the whole blame is imputable, perceived, immediately after the publication of the laft Number, the impropriety of inserting Dr. Nisbet's Case, which introduced the names of regular Practitioners in that form. He therefore, early in October, wrote a Note to Dr. N. of whom he had not the slightest knowledge, requesting an account of the prefent state of his Patient, and his Plan of Treatment. The following is his answer, and the Public will observe how far it is from being satisfactory.*

ST. JAMES'S STREET, 26th *October*, 1800.

"Dr. Nifbet returns Compliments to Dr. Bradley.—The Patient "has been feen fince Dr. B. wrote his Card by Dr. Garthfhore, by "Mr. Blair, furgeon, by Dr. A Buchan, and by Mr. Taylor, "Great Ruffell Street, who have defired Dr. Nifbet to ufe their "names in anfwer to the firft part of his Card.

"On the means of cure, Dr. Nifbet has given a fhort hint in the "ftatement of the cafe, and a farther illuftration of his principles "will be feen in his appendix to his Treatife on Cancer, being a "letter to Dr. Gregory on the fubject. He has alfo given a pro-"mife of a farther detail, fo foon as ten or twelve cafes fhall be "completed and publifhed. Till then he confiders any obferva-"tions as premature."

*Communications have been received from Meffrs. Watt, Sergeant, Caldicott, Cowley, Waker, a Conftant Reader, &c. which fhall be attended to as foon as poffible.*

